# DNA methylation of insulin signaling pathways is associated with HOMA2-IR in primary myoblasts from older adults

**DOI:** 10.1186/s13395-023-00326-y

**Published:** 2023-10-28

**Authors:** Mark A. Burton, Emma S. Garratt, Matthew O. Hewitt, Hanan Y. Sharkh, Elie Antoun, Leo D. Westbury, Elaine M. Dennison, Nicholas C. Harvey, Cyrus Cooper, Julia L. MacIsaac, Michael S. Kobor, Harnish P. Patel, Keith M. Godfrey, Karen A. Lillycrop

**Affiliations:** 1 Human Development and Health Academic Unit, Faculty of Medicine, University of Southampton, Southampton, UK; 2 NIHR Southampton Biomedical Research Centre, University of Southampton & University Hospital Southampton NHS Foundation Trust, Southampton, UK; 3 Biological Sciences, University of Southampton, Southampton, UK; 4 MRC Lifecourse Epidemiology Centre, University of Southampton, Southampton, UK; 5 NIHR Oxford Biomedical Research Centre, University of Oxford, Oxford, UK; 6 Department of Medical Genetics, Faculty of Medicine, Edwin S.H. Leong Healthy Aging Program, University of British Columbia, Vancouver, Canada; 7 Faculty of Medicine, Academic Geriatric Medicine, University of Southampton, Southampton, UK

**Keywords:** Insulin resistance, HOMA2-IR, Skeletal muscle, DNA methylation

## Abstract

**Background:**

While ageing is associated with increased insulin resistance (IR), the molecular mechanisms underlying increased IR in the muscle, the primary organ for glucose clearance, have yet to be elucidated in older individuals. As epigenetic processes are suggested to contribute to the development of ageing-associated diseases, we investigated whether differential DNA methylation was associated with IR in human primary muscle stem cells (myoblasts) from community-dwelling older individuals.

**Methods:**

We measured DNA methylation (Infinium HumanMethylationEPIC BeadChip) in myoblast cultures from vastus lateralis biopsies (119 males/females, mean age 78.24 years) from the Hertfordshire Sarcopenia Study extension (HSSe) and examined differentially methylated cytosine phosphate guanine (CpG) sites (dmCpG), regions (DMRs) and gene pathways associated with HOMA2-IR, an index for the assessment of insulin resistance, and levels of glycated hemoglobin HbA1c.

**Results:**

Thirty-eight dmCpGs (false discovery rate (FDR) < 0.05) were associated with HOMA2-IR, with dmCpGs enriched in genes linked with JNK, AMPK and insulin signaling. The methylation signal associated with HOMA2-IR was attenuated after the addition of either BMI (6 dmCpGs), appendicular lean mass index (ALMi) (7 dmCpGs), grip strength (15 dmCpGs) or gait speed (23 dmCpGs) as covariates in the model. There were 8 DMRs (Stouffer < 0.05) associated with HOMA2-IR, including DMRs within T-box transcription factor (*TBX1*) and nuclear receptor subfamily-2 group F member-2 (*NR2F2*); the DMRs within *TBX1* and *NR2F2* remained associated with HOMA2-IR after adjustment for BMI, ALMi, grip strength or gait speed. Forty-nine dmCpGs and 21 DMRs were associated with HbA1c, with cg13451048, located within exoribonuclease family member 3 (*ERI3*) associated with both HOMA2-IR and HbA1c. HOMA2-IR and HbA1c were not associated with accelerated epigenetic ageing.

**Conclusions:**

These findings suggest that insulin resistance is associated with differential DNA methylation in human primary myoblasts with both muscle mass and body composition making a significant contribution to the methylation changes associated with IR.

**Supplementary Information:**

The online version contains supplementary material available at 10.1186/s13395-023-00326-y.

## Background

Insulin resistance (IR) and impaired insulin secretion are hallmarks of type 2 diabetes mellitus (T2D) [1] and are common in older adults with multiple long-term conditions. Concomitant with an increasingly ageing population, the prevalence of IR and T2D are rising exponentially, with the incidence of T2D amongst older adults currently more than twice that of middle-aged adults [2]. The aetiology of IR and T2D is multifactorial, involving a complex integration of both genetics and environmental exposures [3, 4]. Genetic factors identified by GWAS that influence the pathogenesis of T2D currently explain less than 20% of the heritability [5, 6], with environmental factors that include ageing [7], sedentary behaviour [8, 9], poor nutrition/high-calorie intake [10], obesity [11] and intrauterine environment [12] all causally implicated. Such environmental factors have been suggested to act at least in part through epigenetic processes to modulate IR/T2D risk, with genome-wide DNA methylation analyses showing altered methylation of key regulators of glucose metabolism in both pancreatic islet cells and muscle tissue of individuals with prediabetes and T2D [13, 14].

The skeletal muscle is the primary organ implicated in glucose clearance and is responsible for 80% of postprandial glucose uptake from the circulation [15]. Skeletal muscle IR, which has been reported to be present decades before the onset of β cell failure and symptomatic T2D [15, 16], is caused by desensitization of the muscle to insulin leading to elevated blood glucose levels [16]. During ageing, there are significant changes in body composition with the loss of muscle mass and function (termed sarcopenia) and/or increased adiposity [17]. Both sarcopenia and adiposity lead to structural and functional modifications in the skeletal muscle including impaired mitochondrial bioenergetics, oxidative stress, increased senescence, intramyocellular lipid accumulation, modified activity of insulin sensitivity regulatory enzymes, decreased autophagy and a decline in satellite cell function [18]. Such changes have been suggested to impair the skeletal muscle insulin sensitivity and increase the risk of IR and T2D, although specific underlying mechanisms for increased incidence of T2D in older people have yet to be elucidated.

The skeletal muscle is maintained by muscle stem cells termed satellite cells, which lie beneath the basal lamina, and which become activated (termed myoblasts) and proliferate upon stress or injury; these cells then either differentiate to regenerate or repair muscle fibers or return to a quiescence state to repopulate the satellite cell niche [19]. During ageing, the number and self-renewal capacity of satellite cells have been shown to decline, with alterations in the epigenetic control of quiescence pathways impairing the replenishment of the muscle stem cell pool and contributing to a decreased regenerative response in older age [20–23]. In rodent models of IR/T2D, impaired muscle regeneration has also been observed and this was associated with impaired satellite cell proliferation and myoblast accumulation [24]. Myoblasts from individuals with T2D have also been reported to retain many of the diabetic phenotypes observed in muscle tissue, such as impaired glucose uptake and lipid oxidation [25]. Moreover, differences in DNA methylation have been reported using the 450 K HumanMethylation array in myoblasts from individuals with T2D [26], suggesting epigenetic changes related to T2D are also present in stem cells within the muscle, which may contribute to both impaired myogenesis and regeneration as well as the diabetic phenotype. IR often precedes the onset of T2D, but the relationship between myoblast DNA methylation and IR or the age-related decline in glycaemic control is not known. To gain insight into the underlying gene regulatory mechanisms associated with IR, we used the Infinium Human MethylationEPIC BeadChip (interrogating over 850,000 CpGs sites) to characterise the associations between DNA methylation in human primary myoblasts from community-dwelling older individuals with Homeostatic Model Assessment of Insulin Resistance (HOMA2-IR, an index for the assessment of insulin resistance) and levels of glycated hemoglobin HbA1c, together with the pathways enriched amongst the differentially methylated genes. In addition, we investigated the influence of sarcopenia and adiposity on the HOMA2-IR- and HbA1c-associated methylation signatures to further our understanding of how changes in body composition may contribute to the development of insulin resistance and dysglycaemia in older age.

## Methods

### Study participants

Participants were recruited from the UK Hertfordshire Sarcopenia Study Extension (HSSe), investigating life course influences on muscle function in community-dwelling older people [27]. This study received ethical approval from the Hertfordshire Research Ethics Committee (number 07/Q0204/68) and was conducted in accordance with the 1964 Declaration of Helsinki and its later amendments. Of the 168 HSSe participants, 138 had sufficient DNA from the myoblast cultures for methylation analysis. After quality control of the array data set (outlined below), 119 samples were used for subsequent analyses (Table 1).

**Table 1:** Participant characteristics

Characteristics	Total (*n* = 119)
**Age (years)**	78.24 ± 2.59
**Sex**	Male 30, female 89
**Height (cm)**	163.10 ± 8.27
**Weight (kg)**	72.54 ± 12.10
**BMI (kg/m**^**2**^**)**	27.25 ± 4.06
**Fatmass (kg)**	29.73 ± 8.52
**HOMA2-IR**	1.03 ± 0.58
**Fasting insulin (mU/I)**	7.73 ± 4.42
**HbA1c (mmol/mol)**	37.79 ± 5.33
**Fasting glucose (mmol/l)**	5.43 ± 0.65
**ALMi**	6.23 ± 0.96
**Grip strength**	24.88 ± 9.08
**Gait speed**	0.97 ± 0.20

Values are mean ± standard deviation

### Procedures

Fasting blood samples (overnight) were taken from the anterior cubital fossa, and serum, plasma and fluoride oxalate aliquots were prepared in 1 ml tubes and frozen at − 80℃ until further analyses. Fasting insulin (serum), glucose (plasma) and HbA1c (whole blood) concentrations were measured using Beckman Coulter Access Ultrasensitive Insulin assay (paramagnetic particle, chemiluminescent immunoassay), Beckman Coulter enzymatic UV test (hexokinase method) and Sebia CAPILLARYS HbA1c (separation and quantification of the HbA1c glycated fraction of haemoglobin by capillary electrophoresis), respectively. HOMA2-IR was calculated using the HOMA2 Calculator (v.2.23, University of Oxford) available online at https://www.dtu.ox.ac.uk/homacalculator/. After an overnight fast, percutaneous *vastus lateralis* muscle biopsies (Weil-Blakesley conchotome) containing between 10 and 60 mg of the tissue were conducted under local anesthetic, using a standardised protocol [28], and muscle cell cultures were isolated and frozen down.

### Skeletal muscle stem cell isolation and myoblast culture

Details of muscle stem cell isolation and myoblast culture are described in [23]. Briefly, muscle biopsies were minced and digested in 0.5 mg/mL collagenase (Sigma, UK), before pre-plating to remove fibroblasts. Cells were frozen down at passage 2. Myoblast cultures were then thawed and sorted using CD56 MicroBeads. CD56-positive cells were expanded and plated out for all experiments at passage 4 (P4). Passage 4 cells showed that they were ≥ 96% positive for CD56. Comparison of DNA methylation patterns between HSSe-cultured myoblasts (P4) and muscle biopsies from which the cells were isolated showed a correlation between myoblast and muscle DNA methylation for the 353 Horvath epigenetic clock CpG loci used to determine biological age (*r* = 0.914, *p* ≤ 2.2 × 10^−16^) and for probes within the myogenesis gene set (*r* = 0.960, *p* = 2.0 × 10^−16^) (Supplementary Fig. 1). For this comparison 40 paired HSSe myoblast/muscle samples were assessed, as these were the only muscle biopsy samples where there was sufficient DNA available.

### Infinium Human MethylationEPIC BeadChip array

Genomic DNA (gDNA) was extracted from actively proliferating myoblasts using the GenElute™ Mammalian Genomic DNA miniprep Kit (Merck) following the manufacturer’s instructions. Quality and quantity were determined by Nanodrop (Thermo Scientific) and Qubit™ (Invitrogen). 750 ng of genomic DNA was treated with sodium bisulfite using the Zymo EZ DNA Methylation kit (ZymoResearch, Irvine, California, USA) and hybridised to the Infinium Human MethylationEPIC BeadChip array (Illumina, Inc. CA, USA). This work was carried out at the Centre for Molecular Medicine and Therapeutics (CMMT) (http://www.cmmt.ubc.ca).

### Infinium HumanMethylationEPIC BeadChip array data processing

Myoblast Infinium Human MethylationEPIC data was processed using the Bioconductor package minfi (v1.32.0) [29] in R (v3.6.2). We applied beta-mixture quantile (BMIQ) normalization using the ChAMP package (v.2.16.2) to remove array biases and correct probe design. Methylation profiling was carried out in 142 DNA samples, which included 4 technical replicates. CpGs is known to cross-hybridise to other locations in the genome (*n* = 3027) [30], coinciding with SNPs (*n* = 95,193), with a detection *p* value > 0.01 (*n* = 13,564), and a beadcount < 3 (*n* = 10,368) were removed from the dataset. 16,338 probes aligning to the sex chromosomes and 2815 non-CpG probes were also removed from the dataset. The 4 technical replicate samples were included, for which the Euclidean distance was calculated, and hierarchical clustering, using complete linkage clustered the pairs together. Data were further assessed by visualization of methylation density plots and calculation of median absolute deviation (MAD) scores [31]. The correlation difference for each sample was calculated which is the difference between the average of all the pairwise correlations that involve the sample and the average of all the pairwise correlations that do not. A MAD score (robust *Z* score) is then calculated from the correlation difference by subtracting the medians and dividing it by median absolute deviations (MAD). To determine outliers a standard cutoff (− 5) was set [32]. Technical replicates were removed after normalization but before inference (the duplicate with the lowest MAD score was removed). Twelve samples showed aberrant methylation densities and MAD scores lower than − 5 were removed from subsequent analysis along with 7 samples which had a predicted sex differing to their actual sex. This resulted in 119 samples which were taken forward for further analysis. The methylation data sets analysed generated in this study can be accessed on the gene expression omnibus (https://www.ncbi.nlm.nih.gov/geo/), under accession number GSE221540.

### Infinium Human MethylationEPIC BeadChip array data analysis and statistical analysis

Robust regression models were run using limma (v3.38.3) [33]. Models were adjusted for age, sex, position on array, batch and two surrogate variables (SVs) to account for unknown sources of variation in the data. Surrogate variables (SVs) were calculated using surrogate variable analysis (SVA) [34–36] in bioconductor using all probes. SVs were checked for correlations against phenotypic data to ensure that they were not removing biological data. Both “position on array” and “batch” were used as categorical variables in the linear regression model. Inflation of *p* values was assessed (genomic inflation factor lambda), quantile–quantile (Q-Q) plots generated and *bacon* (v1.10.1) [37] used to control for genomic inflation of test statistics where lambda > 1.2. Since obesity is a major risk factor for T2D, a sensitivity analysis was carried out with additional adjustment for BMI. Analyses took account of multiple tests with the Benjamini–Hochberg adjustment for false discovery rate (FDR), using an FDR < 0.05. Log fold change (logFC) was calculated as part of the linear regression model where the slope is the logFC. Analysis of differentially methylated regions (DMRs) was conducted using DMRcate (v.2.8.5) [38]. DMR significance was ranked by Stouffer score. All statistical analyses were carried out in R (v3.6.2).

### Gene pathway and transcription factor enrichment analysis

Protein–protein interaction (PPI) networks were examined for the top 100 differentially methylated CpG (dmCpG)-associated genes using the Search Tool for the Retrieval of Interacting Genes/Proteins (STRING) and visualized in Cytoscape using a maximum of 2 additional molecules. The top 100 differentially methylated genes were chosen as an arbitrary cutoff to provide sufficient input into the PPI analysis to generate exploratory dmCpG-associated pathways and networks. Pathways were considered if they generated a significant PPI enrichment score (*P* < 0.05) and contained 3 or more genes present within the EWAS top 100. Gene ontology (GO, Biological Process) and KEGG pathway enrichment were determined from the largest network cluster. To investigate upstream regulators and effector networks, ingenuity pathway analysis (IPA) (Qiagen, UK) version 68752261 was used.

### Muscle epigenetic age estimation

Epigenetic age acceleration was calculated as the residuals of regressing the epigenetic age estimated by the Muscle Epigenetic Age Test (MEAT) [39] over chronological age.

## Results

### Participant characteristics

Table 1 shows characteristics of the 119 participants of which 89 were female and 30 male. The mean (SD) age was 78.24 (2.59), body mass index (BMI) (kg/m^2^) 27.25 (4.06), HOMA2-IR 1.03 (0.58), fasting insulin (mU/L) 7.73 (4.42), fasting glucose (mmol/l) 5.43 (0.65) and HbA1c 37.79 (5.33). Of the 119 participants with DNA methylation data, 115 had phenotypic data for HOMA2-IR and 114 for HbA1c. Of the HOMA2-IR characterised participants, 115 had phenotypic data for grip strength, 111 for appendicular lean mass (ALMi) and 115 for gait speed. For HbA1c-characterised participants, 114 had phenotypic data for grip strength, 110 for ALMi and 114 for gait speed.

### Identification of differentially methylated CpGs in myoblasts associated with HOMA2-IR

**Fig. 1:**
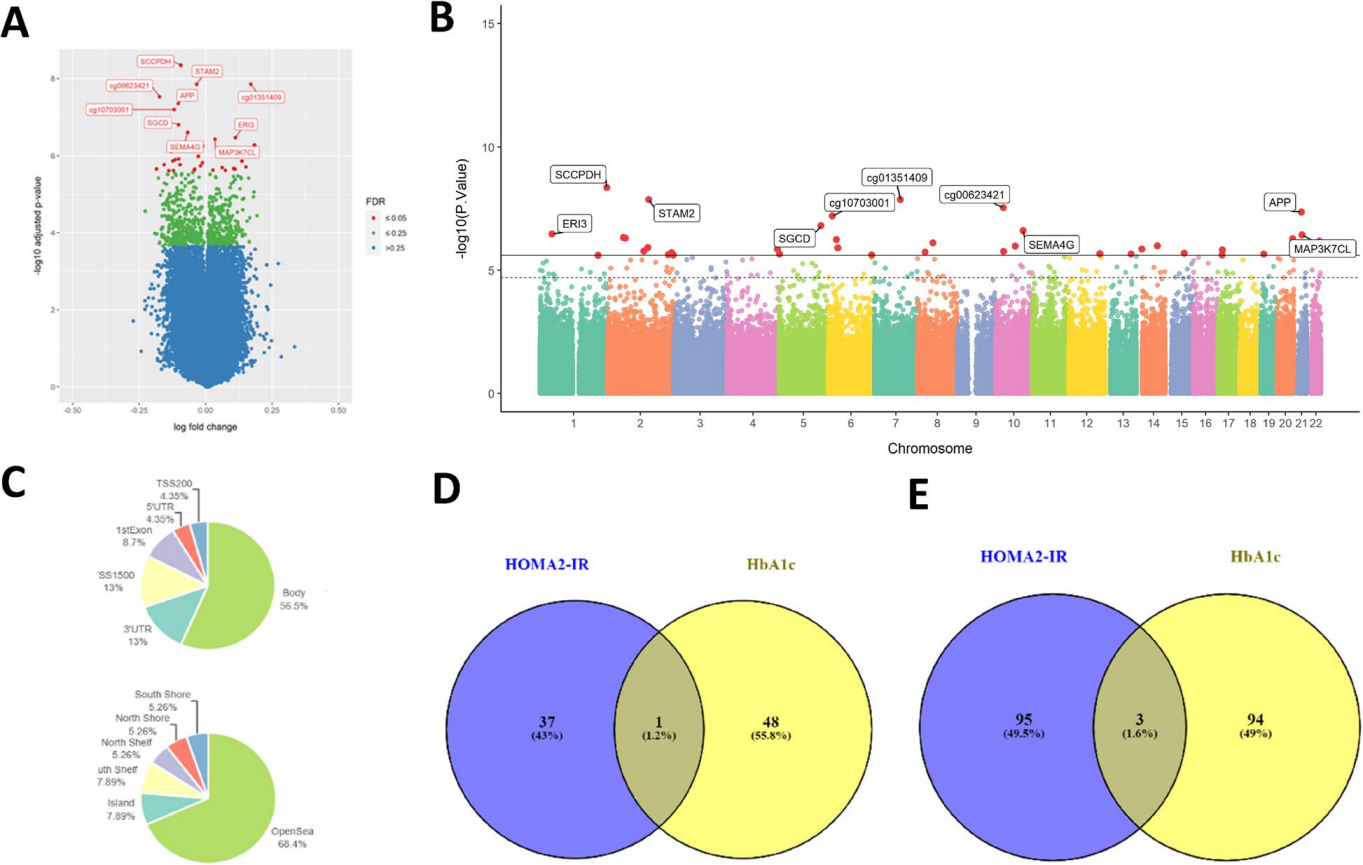
**A** Volcano plot showing the dmCpGs with respect to HOMA2-IR (FDR < 0.05 = red, top 10 FDR < 0.05 labelled). **B** Manhattan plot showing the dmCpGs with respect to HOMA2-IR (FDR < 0.05 = red, top 10 FDR < 0.05 labelled). **C** Locations of dmCpGs with respect to HOMA2-IR (FDR < 0.05). **D** Overlap of dmCpGs (FDR < 0.05) with respect to HOMA2-IR and HbA1c. **E** Overlap of top 100 dmCpG-associated genes with respect to HOMA2-IRand HbA1c

There were 38 differentially methylated CpGs (dmCpGs) significantly (FDR < 0.05) associated with HOMA2-IR (Fig. 1 A, B, C, D, E, Table 2, Supplementary Table 1). The three dmCpGs with the strongest associations were cg08944484, located within the 3′ untranslated region (UTR) of the saccharine dehydrogenase (*SCCPDH*) gene on chromosome 1 (FDR = 3.27 × 10^−03^), cg01351409 located in an intergenic region of chromosome 7 (FDR = 3.37 × 10^−03^) and cg20625334 found within the gene body of signal transducing adapter molecule 2 (*STAM2*) (FDR = 3.37 × 10^−03^).

**Table 2:** HOMA2-IR-associated dmCpGs (FDR < 0.05) in skeletal muscle myoblast cells from elderly individuals

No	Cg ID	logFC	_P	FDR	UCSC Name	UCSC Grp	Location	Chr
1	cg08944484	− 0.093	4.51E-09	0.00327	*SCCPDH*	3′UTR	OpenSea	chr1
2	cg01351409	0.169	1.38E-08	0.00337			OpenSea	chr7
3	cg20625334	− 0.0343	1.40E − 08	0.00337	*STAM2*	Body	OpenSea	chr2
4	cg00623421	− 0.173	2.95E − 08	0.00534			OpenSea	chr10
5	cg19423170	− 0.104	4.42E − 08	0.00641	*APP*	Body	OpenSea	chr21
6	cg10703001	− 0.118	6.36E − 08	0.00769			OpenSea	chr6
7	cg13357714	− 0.102	1.57E − 07	0.0162	*SGCD*	Body	OpenSea	chr5
8	cg15926784	− 0.0682	2.49E − 07	0.0226	*SEMA4G*	1stExon	S_Shelf	chr10
9	cg13451048	0.112	3.40E − 07	0.027	*ERI3*	Body	N_Shore	chr1
10	cg14417798	0.0349	3.72E − 07	0.027	*MAP3K7CL*	1stExon	OpenSea	chr21
11	cg10416784	− 0.0504	4.77E − 07	0.0294			OpenSea	chr2
12	cg17612681	− 0.0395	4.99E − 07	0.0294			OpenSea	chr2
13	cg02219409	0.183	5.28E − 07	0.0294			OpenSea	chr20
14	cg20815084	− 0.0116	5.76E − 07	0.0298	*BAT4*	TSS1500	Island	chr6
15	cg26180255	0.122	6.61E − 07	0.032	*CELSR1*	Body	N_Shelf	chr22
16	cg21097491	0.046	7.47E − 07	0.0334	*FAM53B*	Body	OpenSea	chr10
17	cg04267098	− 0.129	7.84E − 07	0.0334	*PLAG1*	3′UTR	OpenSea	chr8
18	cg11409040	− 0.0275	1.04E − 06	0.04			OpenSea	chr14
19	cg09704075	0.123	1.05E − 06	0.04	*CDH23*	Body	OpenSea	chr10
20	cg02108897	− 0.101	1.23E − 06	0.0434			OpenSea	chr2
21	cg21781422	− 0.114	1.26E − 06	0.0434	*RNF8*	3′UTR	OpenSea	chr6
22	cg11040884	− 0.123	1.38E − 06	0.0436	*PDLIM3*	TSS1500	S_Shore	chr4
23	cg20818795	0.136	1.38E − 06	0.0436			S_Shelf	chr14
24	cg05550692	− 0.0123	1.52E − 06	0.0459	*MED9*	TSS200	Island	chr17
25	cg18584042	− 0.156	1.71E − 06	0.0471	*MIR7853*	Body	OpenSea	chr2
26	cg16072884	− 0.0963	1.72E − 06	0.0471	*KIAA1462*	Body	OpenSea	chr10
27	cg11893955	− 0.0196	1.83E − 06	0.0471			OpenSea	chr8
28	cg08640046	0.151	1.94E − 06	0.0471			S_Shore	chr2
29	cg08148660	0.0619	2.04E − 06	0.0471	*MIR548H4*	Body	N_Shore	chr15
30	cg08379995	0.105	2.16E − 06	0.0471	*RBM19*	Body	OpenSea	chr12
31	cg12331505	− 0.185	2.2E − 06	0.0471			OpenSea	chr5
32	cg12713159	0.11	2.21E − 06	0.0471			OpenSea	chr19
33	cg08800761	− 0.041	2.23E − 06	0.0471	*UGGT2*	Body	OpenSea	chr13
34	cg21554704	0.0275	2.37E − 06	0.0471			OpenSea	chr2
35	cg18185665	0.0743	2.4E − 06	0.0471	*CENPV*	TSS1500	Island	chr17
36	cg07635735	− 0.121	2.42E − 06	0.0471			S_Shelf	chr2
37	cg04349455	− 0.139	2.45E − 06	0.0471	*PARK2*	Body	OpenSea	chr6
38	cg16388983	− 0.0443	2.47E − 06	0.0471	*PTPN14*	5′UTR	N_Shelf	chr1

Log fold change was calculated as the log change in methylation per one unit of HOMA-IR to demonstrate effect size

*LogFC* log fold change, *FDR* false discovery rate, *Chr* chromosome

**Fig. 2:**
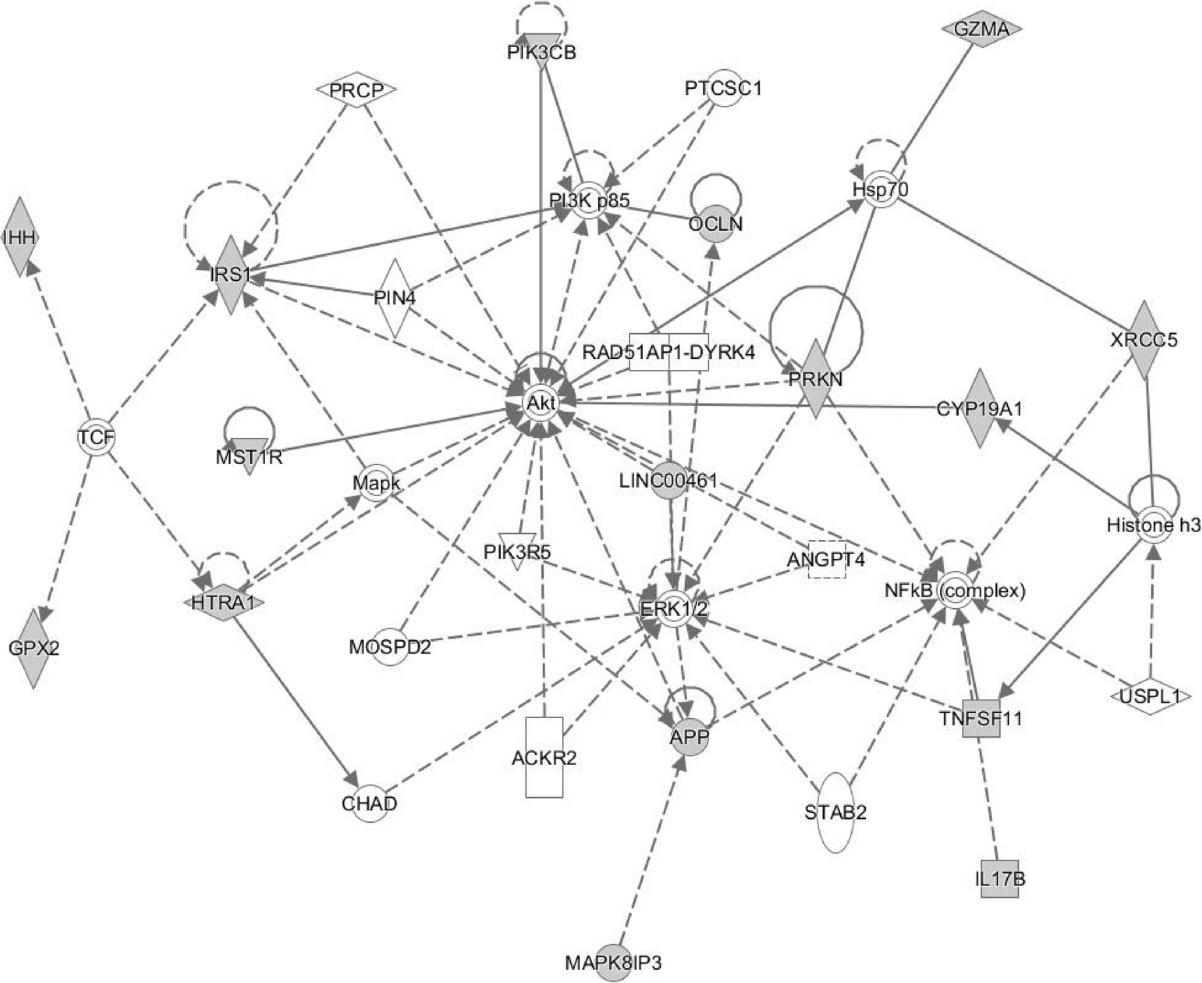
Network diagram showing the top IPA gene network of cellular movement, connective tissue development and function and skeletal and muscular system development and function and associated molecules. Grey nodes indicate molecules within the input dataset, while white nodes are generated by IPA core analysis and represent molecules not in the input dataset added to the network from knowledge base to generate the interaction network

To gain an understanding of the functional significance of the methylation changes associated with HOMA2-IR, the top 100 dmCpG-associated genes were inputted into STRING to generate a PPI network. The PPI enrichment *P* value for the overall network for HOMA2-IR was 4.3 × 10^−02^ indicating biological connection between the proteins. Selection of the largest gene cluster identified within the network was found to be enriched for tissue homeostasis (FDR 6.60 × 10^−04^) anatomical structure homeostasis (FDR 7.50 × 10^−04^) and regulation of JNK cascade (FDR 1.90 × 10^−03^) within gene ontology (GO) pathways for biological process, and AMPK signalling (FDR 1.41 × 10^−02^) and Insulin signalling (FDR 1.41 × 10^−02^) within the KEGG ontology (Table 3, Supplementary Table 2). To identify potential causal networks and upstream regulators that may mediate changes in IR, the HOMA2-IR dmCpG-associated genes were analysed using IPA. The top networks were cellular movement, connective tissue development and function, and skeletal and muscular system development and function (Fig. 2, Supplementary Table 3), with the top upstream regulators identified as T cell factor (TCF) (*p* 3.80E − 04), Clock Circadian Regulator (CLOCK) (*p* 1.58 × 10^−03^) and MAPK (*p* 3.76 × 10^−03^) (Supplementary Table 4).

**Table 3:** HOMA2-IR-enriched pathways in myoblasts from aged skeletal muscle; top 10 Gene Ontology (GO) biological process and top 10 KEGG pathways shown

Genes	Category	Description	FDR	Genes
GO biological process				
7	Bio process	Tissue homeostasis	6.60E-04	*IHH|CTNNB1|OCLN|CDH23|TNFSF11|KIAA1211|ACACA*
8	Bio process	Anatomical structure homeostasis	7.50E-04	*IHH|CTNNB1|OCLN|PARK2|* *CDH23|TNFSF11|KIAA1211|* *ACACA*
6	Bio process	Regulation of JNK cascade	0.0019	*MAPK8IP3|APP|HIPK3|PARK2|* *TNFSF11|AKT1*
9	Bio process	Regulation of MAPK cascade	0.0038	*MAPK8IP3|APP|PIK3CB|MST1R|HIPK3|CTNNB1|PARK2| TNFSF11|AKT1*
12	Bio process	Generation of neurons	0.0038	*SEMA4G|MAPK8IP3|CELSR1|APP|PIK3CB|IHH|GAK|PLAG1|CTNNB1|PARK2|CDH23|AKT1*
10	Bio process	Regulation of transferase activity	0.0038	*MAPK8IP3|APP|PIK3CB|MST1R|HIPK3|IRS1|CTNNB1|PARK2|TNFSF11|AKT1|*
6	Bio process	Regulation of protein kinase b signalling	0.0038	*APP|PIK3CB|MST1R|IRS1|* *TNFSF11|AKT1*
4	Bio Process	Regulation of oxidative stress-induced cell death	0.005	*APP|CTNNB1|PARK2|AKT1*
5	Bio process	Positive regulation of protein kinase b signalling	0.0075	*APP|PIK3CB|MST1R|IRS1| TNFSF11*
12	Bio process	Positive regulation of cell communication	0.008	*MAPK8IP3|APP|PIK3CB|IHH|MST1R|IRS1|C2CD2L|CTNNB1|PARK2|CYP19A1|TNFSF11| AKT1*
KEGG				
4	KEGG	AMPK signalling pathway	0.0141	*PIK3CB|IRS1|AKT1|ACACA*
3	KEGG	Longevity regulating pathway	0.0141	*PIK3CB|IRS1|AKT1*
3	KEGG	Longevity regulating pathway—multiple species	0.0141	*PIK3CB|IRS1|AKT1*
4	KEGG	Insulin signalling pathway	0.0141	*PIK3CB|IRS1|AKT1|ACACA*
3	KEGG	Prolactin signalling pathway	0.0141	*PIK3CB|TNFSF11|AKT1*
3	KEGG	Regulation of lipolysis in adipocytes	0.0141	*PIK3CB|IRS1|AKT1*
5	KEGG	Alzheimer disease	0.0141	*APP|PIK3CB|IRS1|CTNNB1| AKT1*
3	KEGG	Chagas disease	0.0141	*PIK3CB|GNAL|AKT1*
4	KEGG	Hepatitis C	0.0141	*PIK3CB|CTNNB1|OCLN|AKT1*
4	KEGG	Proteoglycans in cancer	0.0141	*PIK3CB|IHH|CTNNB1|AKT1*

*GO* Gene Ontology, *KEGG* Kyoto Encyclopedia of Genes and Genomes

### Influence of BMI on the HOMA2-IR associated DNA methylation signature

To assess the influence of BMI on the HOMA2-IR associated methylation signature in myoblasts, BMI was added as a covariate to the regression model; after adjustment for BMI, there were 6 dmCpGs associated (FDR ≤ 0.05) with HOMA2-IR. The strongest associations were with cg13451048 located within the gene body of exoribonuclease family member 3 (*ERI3)* on chromosome 1 (FDR 3.60 × 10^−03^), and cg01351409 and cg22007860 located in intergenic regions of chromosome 7 (FDR 4.20 × 10^−03)^ and chromosome 3 (FDR 1.40 × 10^−02^), respectively (Supplementary Table 5). Of the 6 dmCpGs associated with HOMA2-IR after adjustment for BMI, 3 overlapped with the dmCpGs identified without adjustment for BMI, these being cg13451048 within *ERI3*, and the intergenic CpGs cg01351409 and cg10416784.

Pathway analysis of the top 100 dmCpG-associated genes after adjustment for BMI showed enrichment of regulation of epidermal growth factor receptor (FDR 1.01 × 10^−02^) and regulation of protein kinase B signalling (FDR 1.89 × 10^−02^) and anatomical structure morphogenesis (FDR 4.06 × 10^−02^) within the GO pathways for biological process, and relaxin signalling (FDR 5.98 × 10^−05^) and endocrine resistance (FDR 2.00 × 10^−04^) amongst the KEGG ontology (FDR 4.00 × 10^−04^) (Supplementary Table 6).

### Influence of sarcopenia on HOMA2-IR-associated DNA methylation signature

As sarcopenia has been implicated in the development of insulin resistance, we adjusted the regression model for each of the 3 definitional components of sarcopenia, namely ALMi, grip strength and gait speed to determine how these measures of muscle mass, strength and function may influence the HOMA2-IR-associated DNA methylation signal in myoblasts (Supplementary Tables 7, 8 and 9). After addition of ALMi as a covariate in the model, the number of dmCpGs associated with HOMA2-IR was reduced from 38 to 7. The top dmCpGs were cg01351409, an intergenic CpG located on chromosome 7, cg18292904 located within the macrophage-stimulating protein receptor gene (*MSTR1*, also known as RON kinase) and cg14878421 an intergenic CpG located on chromosome 4 (Supplementary Table 7). Only 5 of the 38 dmCpGs identified in the unadjusted analysis remained significantly associated with HOMA2-IR; these were cg20625334 (*STAM2*), cg13451048 (*ERI3*) and the intergenic CpGs: cg01351409, cg10703001 and cg00623421, which all showed the same direction of association, effect size and significance. Adjustment for grip strength in the model also attenuated the methylation signal associated with HOMA2-IR with 15 dmCpGs associated with HOMA2-IR after adjustment; 12 of which overlapped with the dmCpGs from the unadjusted analysis (Supplementary Table 8), while the addition of gait speed to the model resulted in the association of 23 dmCpGs with HOMA2-IR, of which 18 of the dmCpGs overlapped with those identified in the unadjusted analyses (Supplementary Table 9).

PPI analysis revealed that the top pathways enriched amongst biological process GO category were regulation of protein kinase B signalling (FDR 2.20 × 10^−05^) and regulation of protein phosphorylation (FDR 4.78 × 10^−05^) after adjustment for ALMi (Supplementary Table 10), regulation of protein kinase B signalling (FDR 2.00 × 10^−03^) and positive regulation of protein kinase B signalling (FDR 4.00 × 10^−03^) after adjustment for grip strength (FDR 2.00 × 10^−03^) (Supplementary Table 11), and generation of neurons (FDR 1.60 × 10^−03^) and positive regulation of kinase activity (FDR 1.90 × 10^−03)^ after adjustment for gait speed (Supplementary Table 12). The top KEGG pathways were MAPK (FDR 8.30 × 10^−05^) and insulin signalling (FDR 8.30 × 10^−05^) after adjustment for ALMi, Prolactin Signalling (FDR 2.90 × 10^−03^) and longevity regulation (FDR 1.34 × 10^−02^) after adjustment for grip strength, and insulin signalling (FDR4.70 × 10^−03^) and bacterial invasion of epithelial cells (FDR 4.70 × 10^−03^) after adjustment for gait speed.

### HbA1c was associated with differential DNA methylation of skeletal muscle myoblasts

To identify methylation changes associated with longer term glycemia, we examined associations between DNA methylation and HbA1c levels. Forty-nine dmCpGs were associated with HbA1c (FDR < 0.05) (Supplementary Table 13), with the top 3 CpGs being cg19477361 located in a CpG island and 5′UTR region of guanine nucleotide-binding protein subunit gamma-7 (*GNG7*) (FDR 2.42 × 10^−03^), cg13451048 located in the gene body of exoribonuclease family member 3 (*ERI3*) on chromosome 1 (FDR 3.06 × 10^−03^), and cg22337620 located in the gene body of the *DLGAP1* gene which encodes for disk large-associated protein 1 (DAP-1), also known as guanylate kinase-associated protein (GKAP) (FDR 3.81 × 10^−03^). Only one dmCpG associated with both HOMA2-IR and HbA1c, which was cg13451048 within *ERI3* (Fig. 1D, Supplementary Table 14). After addition of BMI in the regression model, there were 48 dmCpGs associated with HbA1c (Supplementary Table 15), with 36 of the dmCpGs overlapping with the dmCpGs in the unadjusted model. The addition of ALMi as a covariate resulted in 44 dmCpGs associated with Hb1Ac, with 18 dmCpGs in common with those from the unadjusted analysis (Supplementary Table 16), while the addition of grip strength resulted in 70 dmCpGs associated with Hb1Ac levels with 37 in common with the unadjusted analyses (Supplementary Table 17); adjustment for gait speed led to 64 dmCpGs associated with HbA1c, with 38 overlapping with those identified in the unadjusted analysis (Supplementary Table 18).

Network analysis of HbA1c dmCpG-associated genes showed a PPI enrichment *P* value for the overall network of 1.38 × 10^−02^. Within the largest cluster the top GO biological process pathways were nervous system development (FDR 1.27 × 10^−05^), multicellular organism development (FDR 4.23 × 10^−05^) and cell differentiation (FDR 4.23 × 10^−05^), whilst in the KEGG ontology top pathways were Hippo signalling (FDR 1.60 × 10^−03^) and pathways in cancer (FDR 1.60 × 10^−03^) (Supplementary Table 19). The top pathways enriched within the biological process category, after the addition of BMI as a covariate, were cell communication (FDR 3.79 × 10^−06^), Signalling (FDR 3.79 × 10^−06^) and developmental process (FDR 3.79 × 10^−06^) (Supplementary Table 20). Top pathways enriched were neuron migration (FDR 8.17 × 10^−05^), cell communication (FDR 8.17 × 10^−05^) and signal transduction FDR 8.17 × 10^−05^) after addition of ALMi in the model (Supplementary Table 21); enzyme-linked receptor protein signalling pathways (FDR 4.90 × 10^−03^), cell differentiation (FDR 4.90 × 10^−03^) and signaling (FDR = 5.50 × 10^−03^) after adjustment for grip strength (Supplementary Table 22), and cell differentiation (FDR 8.98 × 10^−06^), generation of neurons (FDR 8.98 × 10^−06^) and cell communication (FDR 1.10 × 10^−04^) after adjustment for gait speed (Supplementary Table 23). Similar KEGG pathways were enriched in both the unadjusted and adjusted analyses.

### HOMA2-IR and HbA1c are associated with multiple differentially methylated regions

Regional analysis identified DMRs associated with HOMA2-IR and HbA1c (Table 4, Supplementary Tables 24 and 25). 8 DMRs were associated with HOMA2-IR (Stouffer < 0.05), with the top 3 DMRs located within the Homobox A3 (*HOXA3)* gene, consisting of 8 CpGs (Stouffer 9.70 × 10^−03^), the T-box transcription factor (*TBX1*) gene consisting of 4 CpGs (Stouffer 1.0 × 10^−03^) and the nuclear receptor subfamily 2 group F member 2 (*NR2F2*) consisting of 4 CpGs (Stouffer 1.3 × 10^−02^), respectively (Table 4). After adjustment for BMI, 12 DMRs were associated with HOMA2-IR, with the top 3 located in *CTD-2562J15.6*, *TBX1* and *N2RF2*, which were all associated with HOMA2-IR in the unadjusted analysis (Supplementary Table 26) Adjustment for each of the definitional components of sarcopenia resulted in 4 DMRs associated with HOMA2-IR; with DMRs being located within *TBX1*, *RNF126P1*, *HOXA3* and *NR2F2* after adjustment for either grip strength or gait speed (Supplementary Tables 27 and 28), and DMRs within NR*2F2*, *RNF126P* and *TBX1* together with an intergenic region of Chr 20 after adjustment for ALMi (Supplementary Table 29). HbA1c was associated with 21 DMRs, with the top DMR located in the HLA Complex Group 9 (*HGC9*) gene (Stouffer 1.9 × 10^−04^) (Supplementary Table 25). There were no DMRs that overlapped between HOMA2-IR and HbA1c. There were 32 DMRs associated with HbA1c after adjustment for BMI (Supplementary Table 30), and 9 after adjustment for either grip strength or gait speed (Supplementary Tables 31 and 32), the top DMR in these analyses being located within HCG9 which is also the top DMR in the unadjusted analysis. Twenty-four DMRs were associated with HbA1c after adjustment for ALMi, the top DMR being located within FERM domain containing 4A (*FRMD4A*) (Supplementary Table 33).

**Table 4:** HOMA2-IR-associated DMRs and overlapping genes (Stouffer < 0.05) in skeletal muscle myoblast cells from elderly individuals

No	Chr	Start	End	Width	no.CpGs	Stouffer	Mean diff	Overlapping genes
1	chr7	27153212	27153944	733	8	0.00970	0.108795	*HOXA-AS2*,* HOXA3*
2	chr22	19754125	19754815	691	4	0.01083	0.152326	*TBX1*
3	chr15	96877194	96877855	662	4	0.01376	0.102643	*NR2F2*
4	chr11	2420649	2420968	320	2	0.02802	0.053311	*NA*
5	chr19	16396929	16397014	86	2	0.02978	0.127776	*CTD-2562J15.6*
6	chr2	2.12E + 08	2.12E + 08	53	3	0.03948	0.057125	*ERBB4*
7	chr13	1.13E + 08	1.13E + 08	357	2	0.04413	0.022587	*ATP11A*
8	chr6	74501269	74501298	30	3	0.04510	− 0.13303	*CD109*

### DmCpGs associated with HOMA2-IR or HbA1c were not related to accelerated epigenetic ageing in skeletal muscle myoblasts

To determine whether the methylation changes associated with HOMA2-IR or HbA1c represented accelerated epigenetic ageing within skeletal muscle myoblasts, epigenetic age was calculated [39]. Epigenetic age as determined by the muscle epigenetic age estimator MEAT was strongly correlated with chronological age (*P* = 0.002), but there were no associations between accelerated epigenetic age and HOMA2-IR (*P* = 0.556) or HbA1c (*P* = 0.552) unadjusted, or after adjustment for BMI, ALMi, grip strength or gait speed (Supplementary Table 34).

## Discussion

Our analyses have shown differential methylation of CpGs in human primary myoblasts from older individuals with respect to HOMA2-IR and HbA1c. The HOMA2-IR-associated dmCpGs were enriched in genes associated with insulin and AMPK signalling pathways, as well as muscle development and tissue homeostasis. The IR-associated methylation signal was attenuated after adjustment for ALMi or BMI, suggesting that muscle mass and body composition make a significant contribution to the methylation changes associated with insulin resistance in myoblasts.

There were changes in DNA methylation in the myoblasts at both the single CpG and regional level associated with HOMA2-IR. Of the top three HOMA2-IR-associated dmCpGs located within a gene, cg08944484 was located within the 3′ untranslated region of the *SCCPDH* gene, which encodes for saccharopine dehydrogenase, a protein which localises to the mitochondrion and is predicted to enable oxidoreductase activity and glycolipid biosynthetic processes [40]; cg20625334 located in the gene body of *STAM2* which encodes a protein that acts downstream of Janus Kinase (JAK) and regulates receptor signalling and trafficking in mammalian cells [41], and cg19423170 located within the *APP* gene which encodes for the amyloid precursor protein. Currently neither *SCCPDH* or STAM2 have been directly linked with IR/T2D although *STAM2* has been shown to be modulated by maternal diabetes in the developing embryo in an animal experimental model [42] suggesting a role for STAM2 in T1D and as a target for perturbations induced by maternal diabetes and transgenerational inheritance of phenotype. The APP protein has been linked with somatic metabolic disorders related to T2D; for example, animal experimental models using *App*^−^/^−^ mice have shown higher levels of insulin degrading enzyme (IDE) mRNA, protein and activity in the skeletal muscle as well as lower fasting levels of blood insulin and a larger increase in response to glucose compared to controls [43]. Mice overexpressing APP protein have further been shown to exhibit mitochondrial dysfunction within the skeletal muscle and a substantial reduction in tricarboxylic acid (TCA) cycle activity indicating a switch from aerobic to anaerobic glucose metabolism [44]. Changes to the methylation status of the *APP* gene associated with IR may indicate a switch in glucose metabolism linked to changes in components of the insulin signalling pathways through modulation of muscle mitochondrial function in older people.

As both sarcopenia and obesity have been implicated in the development of IR, we performed sensitivity analyses additionally adjusting the regression model for the 3 definitional components of sarcopenia and BMI. Adjusting for ALMi attenuated the methylation signature associated with HOMA2-IR suggesting that ALMi makes a significant contribution to the methylation changes associated with insulin resistance in myoblasts. Low muscle mass will impair glucose clearance, and reduce physical inactivity, increasing the risk of IR. Although it has been suggested that there is a bidirectional relationship between IR and sarcopenia, as increased insulin resistance which is characterized by a proinflammatory phenotype and oxidative stress, can impair muscle cell function, leading to losses in the skeletal muscle mass and strength and potentially leading to the development of sarcopenia [45]. A reduction in the HOMA2-IR-associated DNA methylation signal was also observed after addition of grip strength and gait speed as covariates in the analysis. Grip strength is correlated with ALMi and gait speed in this subset of individuals from the HSSe, so the attenuation in the methylation signature after adjustment for grip strength may reflect the lower muscle mass of the individuals.

Increased adiposity is a major risk factor in the development of IR. We found that after adjustment for BMI, 6 dmCpGs were significantly associated with HOMA2-IR, compared to 38 dmCpGs in the unadjusted model, suggesting that body composition contributes to the methylation patterns associated with HOMA2-IR and/or body composition may induce changes in DNA methylation at specific loci, which may in turn influence IR risk. In contrast, dmCpGs not confounded by BMI may be associated with IR via mechanisms independent of body composition. While BMI is only a crude measure of adiposity there was a marked difference in the dmCpGs associated with HOMA2-IR after adjustment for BMI and ALMi with only one dmCpG in common (cg13451048 located within *ERI3*), in accordance with BMI and ALMI reflecting different aspects of body composition.

Pathway analysis revealed that the HOMA2-IR-associated dmCpGs were enriched in genes involved in tissue homeostasis, regulation of JNK, AMPK and insulin signalling pathways. The JNK signalling pathway is involved in stress activation and has increasingly been recognised as an important mediator of IR. For example, animal experimental models have shown that activation of JNK induces IR and ß-cell dysfunction in obesity [46]. While the AMPK signalling pathway also affects insulin action and glucose metabolism; AMPK is an energy sensor which upon activation promotes glucose transport, mitochondrial function, mTOR signalling, fatty acid oxidation and sirtuin expression, while inhibiting inflammation and JNK signalling, oxidative stress and chemokine expression [47]. In model systems and human studies, sustained decreases in AMPK activity induced by obesity or overnutrition accompany IR, whereas AMPK activation is associated with increased insulin sensitivity [48–51]. Differential methylation of genes within the JNK, Insulin and AMPK pathways suggests such changes may contribute to the dysfunction of these pathways leading to IR. Similar pathways were found to be enriched amongst differentially expressed transcripts in muscle tissue from elderly individuals with insulin resistance [52], suggesting epigenetic processes may consolidate or mediate such changes in gene expression resulting in aberrant insulin and downstream signalling perpetuating the insulin-resistant phenotype. After addition of either ALMi, grip strength and gait speed to the model there was enrichment for PKB/AKT signalling pathways, known to play a pivotal role in JNK, AMPK and insulin signalling pathways; this suggests that the epigenetic dysregulation of these pathways in insulin resistant individuals may in part be independent of muscle mass and strength. Interestingly, after adjustment for BMI, although there was still enrichment amongst insulin and JNK signalling pathways, there was also enrichment amongst epidermal growth factor (EGFR) and relaxin signalling pathways suggesting potential effects of IR independent of body composition on muscle stem cell activation, differentiation and survival [53–55].

The top DMRs associated with HOMA2-IR were located within *HOXA3*, *TBX1* and *NR2F2*. *HOXA3* is a DNA binding factor which has been previously reported to be hypermethylation in aged muscle tissue. *TBX1* is a gene that encodes for a member of the T box gene family of DNA binding transcription factors and has been linked to the regulation of insulin signalling and glucose homeostasis within adipose tissue [56]. Within murine muscle *Tbx1* has been shown to modulate muscle fiber type and oxidative metabolism in myotubes as well as stimulate myoblast differentiation [57]. Here, 4 dmCpGs were located within the identified DMR in the gene body or 3′ UTR region of three *TBX1* transcripts. A DMR was also identified within *NR2F2*, also known as COUP transcription factor 2 (COUP-TFII) associated with HOMA2-IR, in both the unadjusted and adjusted analyses. COUP-TFII through its interaction with the glucocorticoid receptor (GR) is known to control gluconeogenesis, through direct binding of COUP-TFII/GR complexes to the promoters of gluconeogenic enzyme genes [58, 59]. In the skeletal muscle of mice, COUP-TFII regulates *Glut4* expression, and in humans, single-nucleotide polymorphisms within the COUP-TFII promoter are associated with insulin sensitivity [59]. Recently in an animal experimental model, it has also been shown that COUP-TFII plays an important role in myogenesis and myoblast fusion, whereby COUP-TFII represses the transcription of Nephronectin (*Npnt*), integrin subunit beta 1 (*Itgb1D*) and Caveolin 3 (*Cav3*), genes important for cell–cell fusion [58]. DMRs within *TBX1* and *NR2F2* remained associated with HOMA2-IR after the addition of BMI, ALMi, grip strength or gait speed as covariates, suggesting these differentially methylated regions are associated with HOMA2-IR independent of body composition, muscle mass or strength. In the BMI-adjusted model, there was also a second DMR containing 10 dmCpG sites identified within *NR2F2*, 8.6 kb downstream of the first DMR; this second DMR overlaps with the *NR2F2-AS1* gene, suggesting that DNA methylation of this region may affect the antisense RNA produced from this locus and the regulation of *NR2F2* through a post-transcriptional mechanism.

DNA methylation was also associated with HbA1c levels; 73% of the dmCpGs associated with HbA1c in the unadjusted analysis overlapped with those in the BMI adjusted analysis, 45% after adjustment for ALMi, 84% after adjustment for grip strength and 78% for gait speed. The dmCpGs associated with HbA1c that were robust to adjustments for BMI, ALMi, grip strength and gait speed included G-protein subunit gamma 7 (*GNG7*), a tumour suppressor gene known to induce autophagy and cell death via the mTOR pathways and inhibit cell division [60] and *ERI3*, a gene involved in RNA processing. The top pathways enriched amongst the HbA1c associated dmCpGs were the nervous system development, cell differentiation, Hippo signalling and pathways in cancer driven by the differential methylation of genes such as BMP7, APC regulator of WNT signalling pathway (APC) and transcription factor 7 like 1 (*TCF7L1*). BMP7 has been reported to increase glucose uptake in muscle by stimulating Glut4 translocation to the plasma membrane through activation of pyruvate dehydrogenase kinase 1 (PDK1) and AKT, suggesting that differential methylation of *BMP7* may affect glucose uptake in myoblasts and potentially blood glucose levels. As insulin resistance is strongly associated with ageing, we investigated whether the dmCpGs associated with HOMA2-IR or HbA1c were associated with accelerated ageing using the MEAT epigenetic clock. We found that although chronological age was strongly related to epigenetic age, the dmCpGs associated with HOMA2-IR or HbA1c were not associated with accelerated ageing, suggesting that although there is an increasing incidence of IR with age, the epigenetic changes associated with IR are distinct from cellular ageing.

Previously there have been limited studies on DNA methylation changes in myoblasts associated with IR. Davegardhl et al. studied differences in DNA methylation in differentiating myoblasts from middle-aged individuals with T2D. They found that pathways involved in steroid biosynthesis, PPAR signalling and biosynthesis of unsaturated fatty acids (USFAs) were upregulated, and the pentose phosphate pathway downregulated upon myoblast differentiation in individuals with T2D compared to normal glucose tolerance (NGT) individuals [26]. In this study, where we compared DNA methylation in actively dividing myoblasts from elderly individuals; we found a significant change in DNA methylation in relation to insulin resistance suggesting DNA methylation changes are present in myoblasts prior to their differentiation into myotubes implying such changes may perpetuate the insulin resistant phenotype.

Strengths of this study are, that to our knowledge, this is the first to examine the association between measures of IR and dysglycemia in aged individuals with DNA methylation in human primary myoblasts. Moreover, we examined the association between DNA methylation and measures of IR across a relatively large number of human primary myoblast cultures isolated from 119 community dwelling older individuals. We were also able to assess the contribution that BMI and measures of muscle mass and strength made to the IR-associated methylation signature. Limitations to the study firstly include the use of cultured human primary myoblasts, where the process of cell culturing may in itself induce epigenetic changes within the cells. However, in the present study, all myoblasts were used at the same early passage number and previous research has demonstrated that cultured primary human myoblasts derived from individuals with T2D retain many of the diabetic phenotypes observed in muscle tissue, such as impaired glucose uptake and lipid oxidation [25]. Secondly, although we carried out the analyses with and without adjustment for BMI, studies have suggested that the visceral fat depot is a major risk factor in the development of IR [61]; further studies investigating the contribution of different fat depots to the HOMA2-IR DNA methylation signal would provide an additional level of understanding of the role of obesity on the methylation changes associated with IR. Thirdly, due to the limited number of early passage cells, we were unable to measure whether the isolated and cultured myoblasts displayed insulin resistance or whether the methylation changes reported here were associated with a corresponding changes in RNA expression or functional changes in the myoblasts themselves. We therefore cannot ascertain whether the changes in CpG methylation play a causal role in the development of IR or are a result of the downstream effects of IR on DNA methylation; however, the demonstration of DNA methylation changes in genes involved in key insulin regulating pathways suggest that they may contribute at least in part to the mechanism by which IR develops and/or is maintained in older skeletal muscle.

## Conclusions

These findings show widespread changes in the methylome of human myoblasts that are associated with HOMA2-IR and HbA1c. Furthermore, we show that HOMA2-IR associated dmCpGs were enriched in genes implicated in insulin signalling and skeletal and muscular system development and function, supporting the premise that epigenetic changes may contribute to the age-related decline in glycaemic control and suggesting that it may be possible to ameliorate the impairments in glycaemic control and improve muscle health in old age by developing intervention strategies which reset the epigenetic landscape of myoblasts.

### Supplementary Information

**Additional file 1: Figure S1.** (A) Correlation of the methylation beta values of the Horvath Pan Tissue CpGs and (B) bland altman plot of these CpGs between the muscle tissue and myoblasts. (C) Correlation of the methylation beta values of the CpGs associated with the genes in the myogenesis geneset (https://www.gsea-msigdb.org/gsea/msigdb/cards/HALLMARK_MYOGENESIS) and (D) bland altman plot of these CpGs between the muscle tissue and myoblasts.

**Additional file 2: Supplementary Table 1.** List of HOMA2-IR dmCpGs. **Supplementary Table 2.** Top 20 GO and KEGG terms associated with the HOMA2-IR networks. **Supplementary Table 3.** IPA Networks from top 100 dmCpG associated genes with HOMA2-IR.** Supplementary Table 4.** IPA upstream regulators from top 100 dmCpG associated genes with HOMA2-IR. **Supplementary Table 5.** List of HOMA2-IR dmCpGs after adjustment for BMI. **Supplementary Table 6.** Top 20 GO and KEGG terms associated with the HOMA2-IR networks after adjustment for BMI. **Supplementary Table 7.** List of HOMA2-IR associated dmCpGs after adjustment for ALMi. **Supplementary Table 8.** List of HOMA2-IR associated dmCpGs after adjustment for grip strength. **Supplementary Table 9.** List of HOMA2-IR associated dmCpGs after adjustment for gait speed. **Supplementary Table 10.** Top 20 GO and KEGG terms associated with the HOMA2-IR networks after adjustment for ALMi. **Supplementary Table 11.** Top 20 GO and KEGG terms associated with the HOMA2-IR networks after adjustment for grip strength. **Supplementary Table 12.** Top 20 GO and KEGG terms associated with the HOMA2-IR networks after adjustment for gait speed. **Supplementary Table 13.** List of HbA1C associated dmCpGs. **Supplementary Table 14.** Common Overlapping dmCpGs. **Supplementary Table 15.** List of fasting HbA1c associated dmCpGs after adjustment for BMI. **Supplementary Table 16.** List of hbA1c associated dmCpGs after adjustment for ALMi. **Supplementary Table 17.** List of hbA1c associated dmCpGs after adjustment for grip strength. **Supplementary Table 18.** List of hbA1c associated dmCpGs after adjustment for gait speed. **Supplementary Table 19.** Top 20 GO and KEGG terms associated with the HbA1c networks. **Supplementary Table 20.** Top 20 GO and KEGG terms associated with the HbA1c networks after adjustment for BMI. **Supplementary Table 21.** Top 20 GO and KEGG terms associated with the hbA1c networks after adjustment for ALMi. **Supplementary Table 22.** Top 20 GO and KEGG terms associated with the hbA1c networks after adjustment for grip strength. **Supplementary Table 23.** Top 20 GO and KEGG terms associated with the hbA1c networks after adjustment for gait speed. **Supplementary Table 24.** HOMA2-IR asscoiated differentially methylated regions. **Supplementary Table 25.** Hba1c asscoiated differentially methylated regions. **Supplementary Table 26.** HOMA2-IR asscoiated differentially methylated regions adjusted for BMI. **Supplementary Table 27.** HOMA2-IR asscoiated differentially methylated regions after adjustment for grip strength. **Supplementary Table 28.** HOMA2-IR asscoiated differentially methylated regions after adjustment for gait speed. **Supplementary Table 29.** HOMA2-IR asscoiated differentially methylated regions after adjustment for ALMi. **Supplementary Table 30.** HbA1c asscoiated differentially methylated regions after adjustment for BMI. **Supplementary Table 31.** HbA1c asscoiated differentially methylated regions after adjustment for grip strength. **Supplementary Table 32.** HbA1c asscoiated differentially methylated regions after adjustment for gait speed. **Supplementary Table 33.** HbA1c asscoiated differentially methylated regions after adjustment for ALMi. **Supplementary Table 34.** MEAT methylation clock for HOMA2-IR and HbA1c unadjusted and adjusted for BMI.

## Data Availability

The methylation data sets generated in this study are available on the gene expression omnibus (https://www.ncbi.nlm.nih.gov/geo/), under accession number GSE221540.

## Abbreviations

IR: Insulin resistance
HSSe: Hertfordshire Sarcopenia Study Extension
dmCpG: Differentially methylated cytosine phosphate guanine
DMRs: Differentially methylated regions
HOMA2-IR: Homeostatic model assessment of insulin resistance
HbA1c: Glycosylated hemoglobin, type A1C
FDR: False discovery rate
JNK: C-Jun N-terminal kinase
AMPK: 5′ AMP-activated protein kinase
BMI: Body mass index
ALMi: Appendicular Lean Mass Index
*TBX1*: T-box transcription factor
*NR2F2*: Nuclear receptor subfamily 2 group F member 2
*ERI3*: Exoribonuclease family member 3
T2D: Type 2 diabetes mellitus
GWAS: Genome-wide association studies
gDNA: Genomic DNA
BMIQ: Beta-mixture quantile
DMEM: Dulbecco’s modified Eagle’s medium
MAD: Median absolute deviation
SVs: Surrogate variables
Q-Q: Quantile-quantile
PPI: Protein–protein interaction
STRING: Search Tool for the Retrieval of Interacting Genes/Proteins
GO: Gene Ontology
IPA: Ingenuity pathway analysis
MEAT: Muscle epigenetic age test
UTR: 3′ Untranslated region
*SCCPDH*: Saccharine dehydrogenase
*STAM2*: Signal transducing adapter molecule 2
TCF: T cell factor
CLOCK: Clock circadian regulator
MAPK: Mitogen-activated protein kinase
*MSTR1*: Macrophage-stimulating protein receptor
*GNG7*: Guanine nucleotide-binding protein subunit gamma-7
DAP-1: Disks large-associated protein 1
GKAP: Guanylate kinase-associated protein
*HOXA3*: Homobox A3
*RNF126P1*: Ring finger protein 126 pseudogene 1
*HGC9*: HLA complex group 9
*FRMD4A*: FERM domain containing 4A
JAK: Janus kinase
*APP*: Amyloid beta precursor protein
T1D: Type 1 diabetes
IDE: Insulin degrading enzyme
TCA: Tricarboxylic acid
PKB/AKT: Protein kinase B
EGFR: Epidermal growth factor
*COUP-TF11*: Nuclear receptor subfamily 2 group F member 2
GR: Glucocorticoid receptor
*Npnt*: Nephronectin
*Itgb1D*: Integrin subunit beta 1
*Cav3*: Caveolin 3
GNG7: G-protein subunit gamma 7
APC: APC regulator of WNT signalling pathway
*BMP7*: Bone morphogenetic protein 7
*APC*: APC regulator of WNT signalling pathway
*TCF7L1*: Transcription factor 7 like 1
Glut4: Glucose transporter type 4
PDK1: Pyruvate dehydrogenase kinase 1
USFAs: Unsaturated fatty acids
NGT: Normal glucose tolerance
