# Supplementary material for: DNA methylation of insulin signaling pathways is associated with HOMA2-IR in primary myoblasts from older adults
Source: Skelet Muscle. 2023 Oct 28;13:17. doi: 10.1186/s13395-023-00326-y (PMC10612387; doi:10.1186/s13395-023-00326-y)
Supplement: Supplementary file 1 — Additional file 1: Figure S1. (A) Correlation of the methylation beta values of the Horvath Pan Tissue CpGs and (B) bland altman plot of these CpGs between the muscle tissue and myoblasts. (C) Correlation of the methylation beta values of the CpGs associated with the genes in the myogenesis geneset (https://www.gsea-msigdb.org/gsea/msigdb/cards/HALLMARK_MYOGENESIS) and (D) bland altman plot of these CpGs between the muscle tissue and myoblasts. [file 13395_2023_326_MOESM1_ESM.docx]

Supplementary Figures


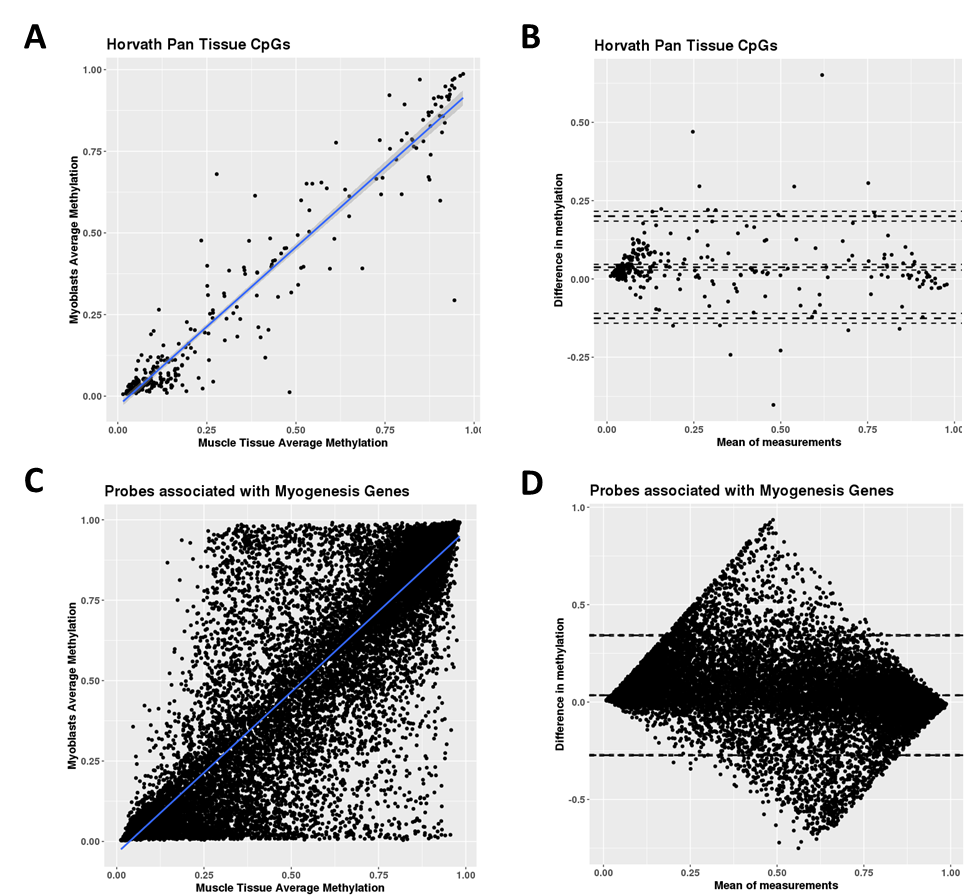


Supplementary Figure 1: (A) Correlation of the methylation beta values of the Horvath Pan Tissue CpGs and (B) bland altman plot of these CpGs between the muscle tissue and myoblasts. (C) Correlation of the methylation beta values of the CpGs associated with the genes in the myogenesis geneset (https://www.gsea-msigdb.org/gsea/msigdb/cards/HALLMARK_MYOGENESIS) and (D) bland altman plot of these CpGs between the muscle tissue and myoblasts.
